# Supplementary material for: Role of invasive Aedes (Aedimorphus) vittatus in chikungunya virus transmission in the newly-invaded coastal island city of Mombasa, Kenya
Source: PLoS Negl Trop Dis. 2025 Dec 9;19(12):e0013738. doi: 10.1371/journal.pntd.0013738 (PMC12707656; doi:10.1371/journal.pntd.0013738)
Supplement: Attachment — Submitted filename: Response to reviewers.docx [file pntd.0013738.s003.docx]

28 September 2025

PLOS Neglected Tropical Diseases

Dear Editor**,**

**Re: Response to Reviewer Comments and Suggestions**

Thank you for the opportunity to revise and resubmit our manuscript. I have carefully considered all the comments and suggestions provided by the reviewers, and we appreciate the constructive feedback. In response, I have made the necessary revisions to the manuscript and addressed each concern raised by the reviewers in detail. Below, we provide a point-by-point response outlining the changes made and the rationale behind them.

1) Please provide an Author Summary. This should appear in your manuscript between the Abstract (if applicable) and the Introduction, and should be 150-200 words long. The aim should be to make your findings accessible to a wide audience that includes both scientists and non-scientists. Sample summaries can be found on our website under Submission Guidelines:
   <https://journals.plos.org/plosntds/s/submission-guidelines#loc-parts-of-a-submission>

**The concern addressed kindly refer to line 44-58 of the clean copy**

2) We have noticed that you have uploaded Supporting Information files, but you have not included a list of legends. Please add a full list of legends for your Supporting Information files after the references list.

**This has been addressed kindly refer to line 690 of the clean copy**

3) When completing the data availability statement of the submission form, you indicated that you will make your data available on acceptance. We strongly recommend all authors decide on a data sharing plan before acceptance, as the process can be lengthy and hold up publication timelines. Please note that, though access restrictions are acceptable now, your entire data will need to be made freely accessible if your manuscript is accepted for publication. This policy applies to all data except where public deposition would breach compliance with the protocol approved by your research ethics board. If you are unable to adhere to our open data policy, please kindly revise your statement to explain your reasoning and we will seek the editor's input on an exemption. Please be assured that, once you have provided your new statement, the assessment of your exemption will not hold up the peer review process.

**This is well noted and I have rephrased the statement as the dataset generated and analyzed in this study will be available in the manuscript, supplementary materials and the sequences will be deposited in gene bank.**

**Reviewers' comments:**

Reviewer's Responses to Questions

**Key Review Criteria Required for Acceptance?**

As you describe the new analyses required for acceptance, please consider the following:

**Methods:**

-Are the objectives of the study clearly articulated with a clear testable hypothesis stated?

-Is the study design appropriate to address the stated objectives?

-Is the population clearly described and appropriate for the hypothesis being tested?

-Is the sample size sufficient to ensure adequate power to address the hypothesis being tested?

-Were correct statistical analysis used to support conclusions?

-Are there concerns about ethical or regulatory requirements being met?

Reviewer #1: Dear Authors,

The submitted manuscript is a result of impressive work that authors conducted in Mombasa. The results represent a significant contribution to the science and are applicable to the regular mosquito control strategies. From the epidemiological point of view obtained results are very useful in the fight against the diseases that are transmitted by identified vectors.
The language of the manuscript is understandable and should not be majorly revised.
However, there are many technical mistakes that have to be corrected before MS is published.
In the whole manuscript, but particularly in introduction, when the species is mentioned for the first time, the author and year of species description should be given.

**This has been addressed kindly refer to line number 85-100 of the clean copy**

Considering that the authors are talking about vector-borne diseases, the overview of the situation in Mombasa and Kenya should be given. Please give the exact numbers of human cases and epidemiological situation.

**Addressed kindly refer to line 69-72 of the clean copy**

In the title, authors are focusing to Ae. vitatus, but they highlight the other vectors as well. The title has to be adapted to the story of the whole manuscript. Aedimorphus should be in bracket. Suggestion for the main title is: Role of mosquito vector species in chikungunya virus transmission and characterization of native Aedes vittatus in the newly-invaded coastal island city of Mombasa, Kenya

**Kindly note we are giving out other species as a secondary data. We did not detect the virus from other vectors including the *Aedes aegypti* the primary vector hence the need to provide the distribution of the other vectors in comparison to *Ae. vittatus.***

Please find my specific comments below:

L19 Missing space- December2021

**Addressed kindly refer to line 19 of the clean copy**

L20 Did you mean Biogent instead of Bioagent?

**Addressed kindly refer to line 20 of the clean copy**

L61 Alphavirus should be written with capital letter.

**Addressed kindly refer to line 60 of the clean copy**

L84 Not clear. Please correct - area of Europe Africa, it is

**Addressed kindly refer to line 86 of the clean copy**

L100 Not clear. Did you mean- giving its potential? Please revise the sentence. Same for Line 109.

**Addressed kindly refer to line 107 and 114 -117 of the clean copy**

L120 Please correct- (Figure (Fig) 1). to (Fig. 1) and delete comma before the bracket.

**Addressed kindly refer to line 127 of the clean copy**

L143-144 This is not needed to be given in details - “while another copy was securely stored in a locked cabinet with restricted access in the PI’s office at KEMRI”. It should be shorten to- while another copy was kept in KEMRI.

**Addressed kindly refer to lines 154-155 of the clean copy**

L147 This sentence seems incomplete from the part- and compounds …

**Addressed kindly refer to lines 157-161 of the clean copy**

L147-157 Why did you adapt sampling to the biology of Ae. aegypti and you were sampling Ae. vittatus? You should explain that here or you should change the species in this section.

**Addressed kindly refer to line 164 of the clean copy**

L154 Please correct - Lower part of legs.

**Addressed kindly refer to lines 167-168 of the clean copy**

L163 Missing space before bracket. And space in bracket should be deleted.

**Addressed kindly refer to line 177 of the clean copy**

L176 Years should be deleted. Instead of (≤25 please write up to 25 or maximum 25.

**Addressed kindly refer to line 191 of the clean copy**

L212 Why not in all 843 pools?

**Addressed kindly refer to line 227 of the clean copy**

L264 Please give the citation for this formula.

**Citation for the formula given kindly refer to line 279 0f the clean copy**

L278 Instead- collected with, should be written – of which Ae. aegyptii is having….

**Addressed kindly refer to line 293 0f the clean copy**

L280 …than in the morning… Also space is missing before the bracket.

**Addressed kindly refer to line 295 0f the clean copy**

L281 Please give only two decimals in this line but the same is suggested for the whole manuscript (L192). Here should be – In the BGS

**Addressed kindly refer to line 296 0f the clean copy**

L286 Please delete- summary of

**Addressed kindly refer to line 301 0f the clean copy**

L294 No need to give the abbreviation of locations more than once in the text. Latter abbreviation or full name should be use, not both.

**Addressed kindly refer to lines 324-328 0f the clean copy**

L298 Should be (Fig. 2).

**Addressed kindly refer to line 333 0f the clean copy**

L300 Title should not be in bold. Same for other figures.

**This has been well addressed through out the manuscript, kindly refer to line 353 0f the clean copy**

L306 No need for italics.

**Addressed kindly refer to line 340 0f the clean copy**

L314 Please correct (Supplementary fig1). to (Supplementary 1).

**Addressed kindly refer to line 348 0f the clean copy**

L316 Missing space before the bracket. You should give the exact p value. It should be - significant difference or statistically significant difference.

**The section was descriptive, showing the relative abundance of mosquito species, and therefore did not require a statistical test. The p-value that appeared in this section was inadvertently misplaced and should have been reported in line 322. We acknowledge this error and have corrected it. Kindly refer to line 349 0f the clean copy**

L319 Should be (Fig. 3). Same in L339.

**Addressed kindly refer to lines 352, 371 0f the clean copy**

L331 Instead of community for mosquitoes please use the word population. Please correct accordingly in the whole manuscript.

**Addressed kindly refer to line 363 0f the clean copy**

L339 Missing full stop.

**Addressed kindly refer to line 371 0f the clean copy**

L347 Please delete subtitle because you already gave it above. Same for Beta subtitle afterward.

**Addressed kindly refer to lines 378, 389 0f the clean copy**

L385 Instead of mosquito collection event, it should be mosquito sample. Please correct in the whole manuscript.

**The word event has been changed and well addressed through the manuscript kindly refer to line 398-400 of the clean copy**

L360 Give exact p value. If the p value is so low such as 0.00000005 in that case you should write p<0.001. If it is higher than 0.001 or the same as 0.001, you should write exact number. But also you should give exact statistical parameters for all. This version is not acceptable.

**Given that the statistical data was already highlighted in line 381 to 383 we just opted to put the greater than sign in the figure description to avoid repetition.**

L382 Please fit the title and the figure on one page.

**Addressed kindly refer to line number 406 of the clean copy**

L385-386 The sentence in bold should be deleted and only left in bracket (MSA = Mombasa)

**Addressed kindly refer to lines 409-410 of the clean copy**

L400 Please correct to..... which is expected.... Or give a full stop before „that“.
Figure 8. Correct title. It is not human bite. It is mosquito bite. Please correct that in the whole manuscript. In A graph space is missing in title before bracket. A and B should be separated as two Figures.

**Addressed kindly refer to lines 423, 427 and attached figure 8 of the clean copy**

L408 Missing space after year

**Addressed kindly refer to line 431 of the clean copy**

L415 Citation should be given here.

**Addressed kindly refer to line 438 of the clean copy**

L406 Please delete - identified in the study.
Fig 9 and its title should be fitted in the one page.

**Addressed kindly refer to lines 429 and 440 of the clean copy**

L427 vectors of viruses

**Addressed kindly refer to line 449 of the clean copy**

L434 After CHIKV should be comma.

**Addressed kindly refer to line 456 of the clean copy**

L439-440 Wrong format of citation.

**Addressed kindly refer to line 462 of the clean copy**

L444 This is not correct to say that study confirmed that species is native because of that analyses. The analyses confirmed that the specimens which you analyzed are closely related to those that were before analyzed, also from Kenya.

**Addressed kindly refer to lines 465-467 of the clean copy.**

L453 Missing full stop.

**Addressed kindly refer to line 475 of the clean copy**

L462 Wrong format of citation.

**Addressed kindly refer to line 484 0f the clean copy.**

L468 Delete space - areas ,

**Addressed kindly refer to line 503 of the clean copy**.

References:
What is - Kilcullen D. Mombasa: Gateway to Africa. 2019.? Please add link or more information.

**Addressed kindly refer to line number 630 of the clean copy**

Ref 22 should be corrected. It is WHO or write the full name. Please add the link.
The references require major revision. The space between the lines is not adequate, the name of the species is not in italics and there are technical mistakes such as missing spaces there that require corrections.

**Addressed kindly refer to lines 634, of the clean copy**

Reviewer #2: Yes the objectives are well stated and also the design of the study is good. My only concern is about the ethical approval of carrying out HLC (human landing) collection where Ae. aegypti is involved and there is no cure or medication for dengue. Anyway they have obtained clearance from their Ethics committee

**The study participant are community health workers who well trained on the human landing collection.**

Reviewer #3: (No Response)

**Results:**

-Does the analysis presented match the analysis plan?

-Are the results clearly and completely presented?

-Are the figures (Tables, Images) of sufficient quality for clarity?

Reviewer #1: Given in the text above.

Reviewer #2: The results have been well presented. On the whole it is good.

Reviewer #3: (No Response)

**Conclusions:**

-Are the conclusions supported by the data presented?

-Are the limitations of analysis clearly described?

-Do the authors discuss how these data can be helpful to advance our understanding of the topic under study?

-Is public health relevance addressed?

Reviewer #1: Given in the text above.

Reviewer #2:

The paper is well written. My only concern is that there is no in depth discussion about what can be done to control the Ae. vittatus.

**Addressed kindly refer to line 530-535 of the clean manuscript.**

Does Ae. vittatus breed along with Ae. aegypti in containers? Has it been found when larval surveys were carried out for Ae. aegypti by health teams. I know it is not part of this study but it can be included in the introduction or discussion.

**Addressed kindly refer to lines 450-554 of the clean manuscript.**

The results shows that only one pool of Ae. vittatus was positive - thus it is an important vector. Even Ae. aegypti were not positive. Thus, for the benefit of the readers more information should be provided about Ae. vittatus.

**Addressed kindly refer to line 481-484 of the clean manuscript.**

Reviewer #3: (No Response)

**Editorial and Data Presentation Modifications?**

Use this section for editorial suggestions as well as relatively minor modifications of existing data that would enhance clarity. If the only modifications needed are minor and/or editorial, you may wish to recommend “Minor Revision” or “Accept”.

Reviewer #1: Given in the text above.

Reviewer #2: This is okay

Reviewer #3: (No Response)

**Summary and General Comments:**

Use this section to provide overall comments, discuss strengths/weaknesses of the study, novelty, significance, general execution and scholarship. You may also include additional comments for the author, including concerns about dual publication, research ethics, or publication ethics. If requesting major revision, please articulate the new experiments that are needed.

Reviewer #1: Given in the text above.

Reviewer #2: As i have mentioned above, please provide more details about the breeding of Ae. vittatus.

**Addressed kindly refer to lines 450-554 of the clean manuscript.**

Also provide some methods that can be used by the surveillance team for the control. Most of my comments have been mentioned under the conclusion section.

**Addressed kindly refer to line 530-534 of the clean manuscript.**

Reviewer #3: General Comments

The manuscript by Mulwa et al. examines the abundance, biting activity, and potential role of Aedes vittatus in chikungunya virus (CHIKV) transmission in Mombasa, Kenya. The study is timely, relevant, and contributes valuable entomological and virological data, particularly the detection of CHIKV in Ae. vittatus. However, several interpretations are stronger than the evidence supports, and methodological details require clarification. See my comments below.

Major Comments

1. The high numbers of Ae. vittatus collected across multiple sites are suggestive of establishment in Mombasa. However, firm conclusions that the species has “adapted” and “successfully established” are not yet fully supported.

**Addressed kindly refer to line 485-498 of the clean manuscript.**

No longitudinal data, larval surveys, or host-feeding studies are presented to confirm sustained presence.

**Larval sampling had been conducted in the area as indicated in line of the revised clean copy. For the longitudinal survey I have indicated this as a limitation to the study kindly refer to lines 536-540 of the clean copy**

Moreover, the phylogenetic tree (Fig. 7) shows that the haplotypes from the present study (red) cluster distinctly from earlier Kenyan collections (blue), suggesting possible new introductions or reintroductions rather than continuity of a single established population. These claims should therefore be presented more cautiously, while acknowledging that the observed abundance provides preliminary evidence of establishment.

**Noted and this has been addressed kindly refer to line 465-470 of the clean copy**

2. No parity dissections or age-grading were conducted, limiting assessment of whether Ae. vittatus can survive long enough to sustain CHIKV transmission. This should be acknowledged as a limitation.

**Noted and this has been addressed kindly refer to lines 538-540 of the clean copy**

3. CO₂ baited BG traps operated continuously for 24 h, while HLC was restricted to two 3-h daytime windows. Raw totals are therefore not directly comparable as presented in L290-298.

**This was done to show the two methods can be used to collect the aedes mosquitoes since the HLC was considered as a goal standard. The has been addressed kindly refer to lines 318-328 of the clean copy**

Results should be standardized (e.g., mosquitoes per trap-hour vs. person-hour), and if possible, BG catches should be disaggregated by day vs. night. Otherwise, only within-method comparisons are defensible.

**Noted and this has been addressed kindly refer to line 303 of the clean copy.**

Also, an analysis correlating trap abundance (BG catches) with human biting rates (HLC) would strengthen epidemiological interpretation.

**Noted and this has been addressed kindly refer to lines 499-505 of the clean copy**

4. If collections in June and December could allow for seasonal comparisons, these results should be presented or explicitly noted as absent.

**Noted and this has been addressed kindly refer to lines 485-498 of the clean copy**

5. Figure 3 suggests that most of the Ae. vittatus captured using BG traps may also have been captured at night. This result is not highlighted in the results section.

**Noted and this has been addressed kindly refer to lines 499- 505 of the clean copy**

6. How do the diversity indices sections (L330-367) add to the conclusions of this study?

**Understanding arbovirus transmission dynamics requires knowledge not only of virus detections but also of the mosquito species circulating in an area and their abundance/diversity. The diversity indices therefore support our conclusion that arbovirus transmission is influenced by multiple mosquito species. Concern addressed kindly refer to lines 547-555 of the clean copy**

7. Some aspects of the data presentation and analysis are unclear. For example, in Figure 8A the “average bite rate” appears higher than the rates reported from individual methods, which is difficult to interpret. Similarly, certain outputs (e.g., very low degrees of freedom) suggest results may be based on limited replication (i.e, analysis of total instead of replicate-level data). The authors should clarify their analytical approach for each comparison.

**Noted and this has been addressed kindly refer to lines 417 and 418 of the clean copy and attached figure 8**

6. The opening paragraph of the Discussion section reads more like background. It should begin with a concise summary of the key findings (abundance, biting activity, CHIKV isolation) before contextualizing these results within the broader literature.

**This has been addressed kindly refer to line 447 of the clean copy**

Minor comments

-Improve transitions between paragraphs in the introduction. Currently, paragraphs feel disjointed.

**Noted and this has been addressed kindly refer to lines 73, 85 and 101 of the clean copy**

-Standardize “Biogent” (not “Bioagent”)

**Noted and this has been addressed kindly refer to lines 20,180,315 of the clean copy**

-Clarify the use of “native” versus “invasive” when describing Ae. vittatus. See full title and short title.

**Noted and this has been addressed kindly refer to line 1 of the clean copy**

-State BG trap effort (trap-days) in the methods section.

**Noted and this has been addressed kindly refer to line 184 of the clean copy**

-The HLC description (L147-165) emphasizes Ae. aegypti rather than Ae. vittatus. Clarify the rationale.

**Noted and this has been addressed kindly refer to line 141 of the clean copy**

-Provide details on the study sites.

**Noted and this has been addressed kindly refer to lines 127-133 of the clean copy**

-Clarify which version of R was used for analysis.

**Noted and this has been addressed kindly refer to line 272 of the clean copy**
-Table 1 should include site-level breakdowns.

**Noted and this has been addressed kindly refer to supplementary 1 attached in the manuscript.**

-Avoid presenting the same data in text, tables, and figures.

**Noted and this has been addressed**

-The phrase “multiple presence of the species” L38 is vague and should be clarified.

**Noted and this has been addressed kindly refer to line 38 of the clean copy**

Thank you for your time and consideration.

**Sincerely,**

**Francis Mulwa**
